# Similarities Between Embryo Development and Cancer Process Suggest New Strategies for Research and Therapy of Tumors: A New Point of View

**DOI:** 10.3389/fcell.2019.00020

**Published:** 2019-03-07

**Authors:** Giovanni Manzo

**Affiliations:** General Pathology, “La Sapienza” University of Rome, Retired, Botrugno, Italy

**Keywords:** HSP70, HLA-G, ESCs, MSCs, CSCs, tumor hierarchy/immunoevasion/therapy/prophylaxis

## Abstract

Here, I propose that cancer stem cells (CSCs) would be equivalent to para-embryonic stem cells (p-ESCs), derived from adult cells de-re-programmed to a ground state. p-ESCs would differ from ESCs by the absence of genomic homeostasis. A p-ESC would constitute the cancer cell of origin (i-CSC or CSC0), capable of generating an initial tumor, corresponding to a pre-implantation blastocyst. In a niche with proper signals, it would engraft as a primary tumor, corresponding to a post-implantation blastocyst. i-CSC progeny would form primary pluripotent and slow self-renewing CSCs (CSC1s), blocked in an undifferentiated state, corresponding to epiblast cells; CSC1s would be tumor-initiating cells (TICs). CSC1s would generate secondary CSCs (CSC2s), corresponding to hypoblast cells; CSC2s would be tumor growth cells (TGCs). CSC1s/CSC2s would generate tertiary CSCs (CSC3s), with a mesenchymal phenotype; CSC3s would be tumor migrating cells (TMCs), corresponding to mesodermal precursors at primitive streak. CSC3s with more favorable conditions (normoxia), by asymmetrical division, would differentiate into cancer progenitor cells (CPCs), and these into cancer differentiated cells (CDCs), thus generating a defined cell hierarchy and tumor progression, mimicking somito-histo-organogenesis. CSC3s with less favorable conditions (hypoxia) would delaminate and migrate as quiescent circulating micro-metastases, mimicking mesenchymal cells in gastrula morphogenetic movements. In metastatic niches, these CSC3s would install and remain dormant in the presence of epithelial/mesenchymal transition (EMT) signals and hypoxia. But, in the presence of mesenchymal/epithelial transition (MET) signals and normoxia, they would revert to self-renewing CSC1s, reproducing the same cell hierarchy of the primary tumor as macro-metastases. Further similarities between ontogenesis and oncogenesis involving crucial factors, such as ID, HSP70, HLA-G, CD44, LIF, and STAT3, are strongly evident at molecular, physiological and immunological levels. Much experimental data about these factors led to considering the cancer process as ectopic rudimentary ontogenesis, where CSCs have privileged immunological conditions. These would consent to CSC development in an adverse environment, just like an embryo, which is tolerated, accepted and favored by the maternal organism in spite of its paternal semi-allogeneicity. From all these considerations, novel research directions, potential innovative tumor therapy and prophylaxis strategies might, theoretically, result.

## Introduction

Nearly 30 years ago, I proposed that cancer stem cells (CSCs) would be cells blocked at early steps of their genic program, with reiterated expression of embryonic factors responsible for malignant characters and loss of differentiated factors for terminal genomic homeostasis: thus, CSCs would be equivalent to para-embryonic stem cells (p-ESCs) (Manzo, 1989). The main aspects of stem cells (SCs) are self-renewal, pluripotency (Liu et al., 2007) and the need for a niche (Plaks et al., 2015) with proper stereotrophic factors (space, oxygen) and persistent specific signals (ACTIVIN-A, BMP, WNT, LIF, FGF, TGFb) (Okita and Yamanaka, 2006; Xiao et al., 2006).

**FIGURE 1:**
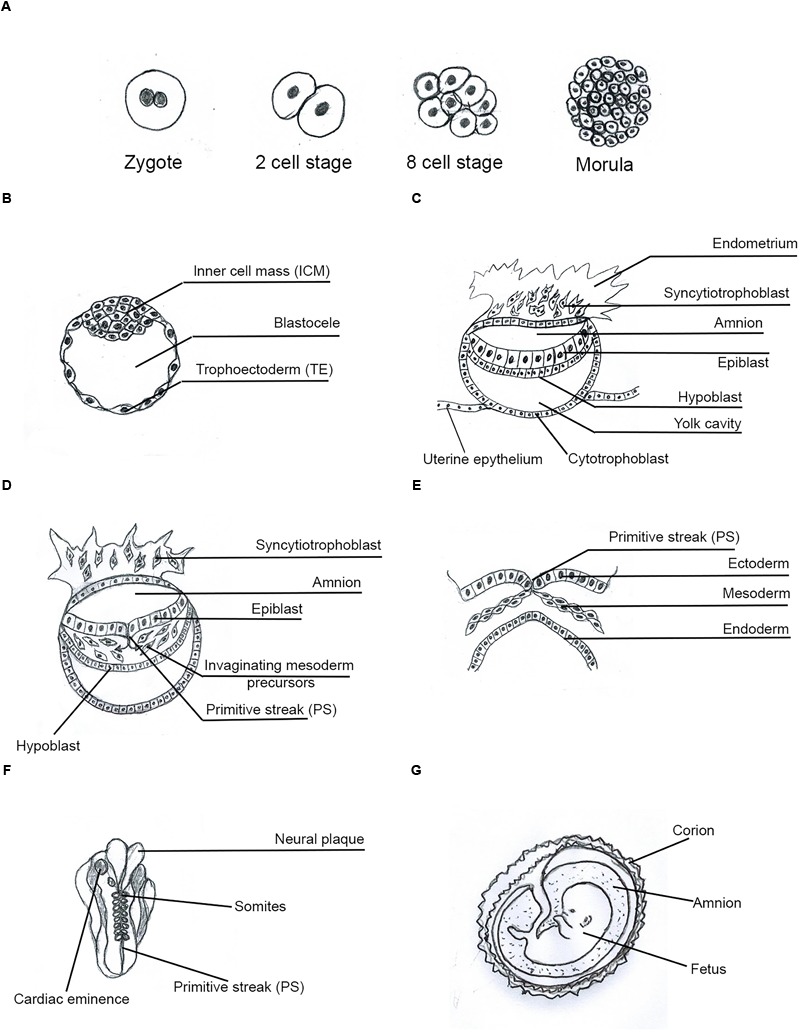
Human Embryo development. Main phases and structures of the embryogenesis process. **(A)** Zygote to morula transition; **(B)** pre-implantation blastocyst; **(C)** implanted blastocyst; **(D)** early gastrula; **(E)** late gastrula; **(F)** somito-histo-organogenesis; **(G)** fetal growth-differentiation.

*ESCs* arise from the inner cell mass (ICM) of mammalian pre-implantation blastocyst (Henderson et al., 2002; Ginisa et al., 2004; Figure 1B); they can self-renew symmetrically and indefinitely, maintain the widest pluripotency and generate all cell lineages of the body. This phenomenon requires defined transcription factors (TFs) specifically expressed in SCs, such as OCT4, SOX2, NANOG, STAT3, KLF4, c-MYC et al., that together constitute a pluripotency gene regulatory network (PGRN) (HaKashyap et al., 2009; Do et al., 2013; Festuccia et al., 2013). Human ESCs (hESCs) and human embryos express comparable stage-specific embryonic antigens (Henderson et al., 2002) and can differentiate into the trophectoderm (TE) by BMP4 (Xu et al., 2002; Figure 1B). hESCs are epithelial cells (Ullmann et al., 2006), but during *in vitro* differentiation they can acquire a mesenchymal phenotype (Eastham et al., 2007).

*MSCs* (mesenchymal stem cells) have a mesenchymal phenotype and markers (Ullmann et al., 2006; Eastham et al., 2007; Thiery et al., 2009). MSCs, in Matrigel, grow at the periphery of hESC clusters, have an undifferentiated phenotype and preserve potential expression of pluripotency TFs such as NANOG and OCT4. This indicates that ESCs can undergo epithelial–mesenchymal transition (EMT) without loss of pluripotency, which would be expressed after mesenchymal–epithelial transition (MET) (Ullmann et al., 2006; Thiery et al., 2009). Cells with mesenchymal features largely lie at the primitive streak (PS) in the embryo and in the tumor stroma (Thiery et al., 2009; Nishimura et al., 2012; Figure 1C).

*CSCs* are tumor cells that are able to generate all the cell types present in the primary tumor and to form metastases, with identical cell types and hierarchy (Marjanovic et al., 2013; Cabrera et al., 2015). CSCs are a small portion of the tumor mass (Collins et al., 2005; Liu et al., 2014) and are often distinct in tumor-initiating cells (TICs) and tumor migrating cells (TMCs) (Hermann et al., 2007; Biddle et al., 2011). TICs have an epithelial phenotype and are able to grow in an anchorage-independent way, to produce spheroids *in vitro* by self-renewal and to initiate tumor development. TMCs have a mesenchymal phenotype, are free, migrating, invasive and generally quiescent, but are able to generate metastases (Dieter et al., 2011; Brabletz, 2012; Liu et al., 2014).

**FIGURE 2:**
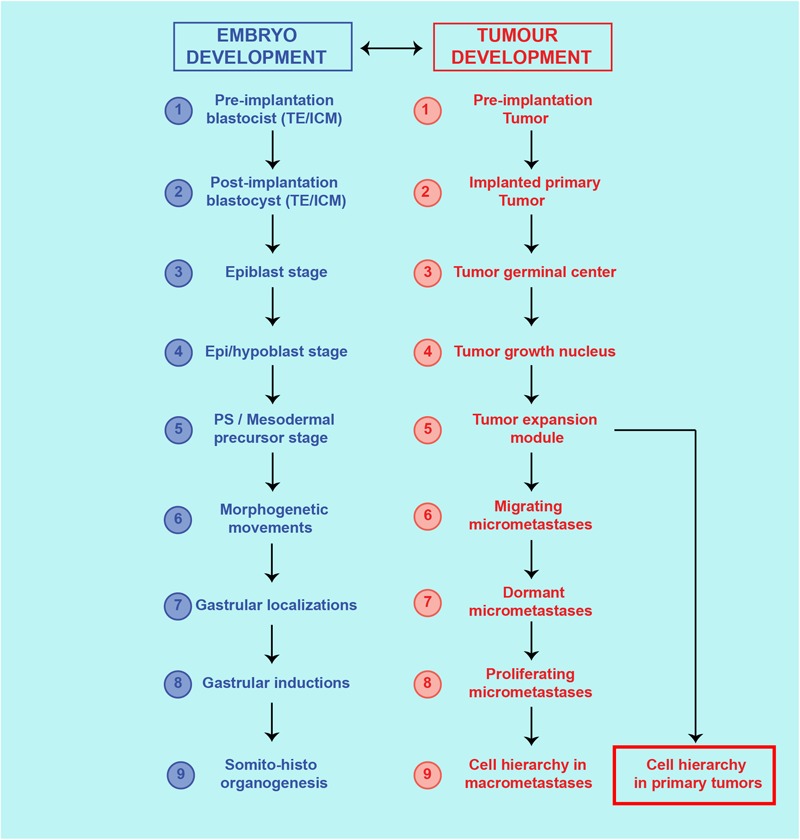
Theoretical similarities between cancer process and ontogenetic development. Correspondence of steps and structures between the cancer process and embryo development.

Therefore, cells with ESC, MSC, and CSC features are at the basis of both embryo development and cancer process (Figure 2).

## The Tumor Process as Ectopic Rudimentary Ontogenesis

### Cell of Origin (CSC0): Initial Cancer Stem Cell (i-CSC) as a Reprogrammed Para-ESC

**FIGURE 3:**
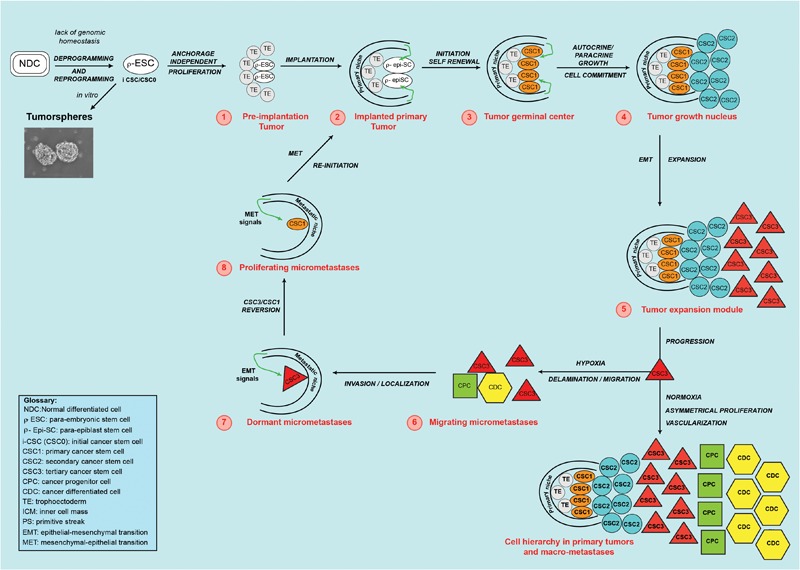
Theoretical cancer process. A normal differentiated cell (NDC), in the absence of genomic homeostasis, would be de-programmed and re-programmed to a para-ESC (p-ESC), constituting the initial cancer stem cell (i-CSC or CSC0). **(1)** An i-CSC, by anchorage-independent proliferation, would generate p-ESC progeny, forming an initial tumor *in vivo* (1-red) (or a tumorsphere *in vitro*), corresponding to a pre-implantation blastocyst (TE/ICM). **(2)** The initial tumor would install in a niche with proper factors and develop a primary tumor, mimicking an implanted blastocyst in the endometrium. **(3)** By PGRN activity, primary undifferentiated, pluripotent, slow self-renewing CSCs (CSC1s/TICs) would arise. CSC1s, epigenetically blocked in a ground/primed state, corresponding to the epiblast state, would form a “tumor germinal center,” continuously feeding the tumor. **(4)** Through autocrine/paracrine growth, from CSC1s, committed non-self-renewing secondary CSCs (CSC2s) would arise. CSC1s and CSC2s, together, would form a “tumor growth nucleus” corresponding to the epiblast/hypoblast state. **(5)** From CSC1s/CSC2s, tertiary CSCs (CSC3s), with a mesenchymal phenotype (EMT), would be generated. CSC3s would be able to migrate and invade adjoining sites, mimicking delamination of mesodermal precursors at the primitive streak. CSC1s, CSC2s, and CSC3s, together, would form a “tumor expansion module”. In the growing primary tumor, favorable conditions (normoxia) would induce CSC3s to proliferate asymmetrically and generate CPCs and, then, CDCs. This would determine a CSC1-CSC2-CSC3-CPC-CDC cell hierarchy and tumor progression, thus mimicking a partial, rudimentary somito-histo-organogenesis process. **(6)** In parallel, unfavorable conditions (hypoxia) would induce CSC3s to migrate as circulating micro-metastases, mimicking the gastrula morphogenetic movements. **(7)** In distant niches with proper EMT signals, CSC3s in the niche would locate as quiescent micro-metastases, mimicking embryonic locations of mesodermal cells at somitogenesis sites. **(8)** Micro-environmental proper MET signals, would induce mesenchymal CSC3 to revert, into epithelial self-renewing CSC1s mimicking gastrular induction (TICs). Self-renewing CSC1s, as TICs, would form a new tumor germinal center and re-initiate the tumor process in metastatic sites, repeating the same steps and reproducing the same cell types and hierarchy of the primary tumor. Tumorsphere figure was adapted from Bond et al. (2013).

Reprogramming would be the main mechanism for genesis and proliferation of the initial i-CSC (CSC0): it has been shown that somatic cell reprogramming requires a MET at its initiation (Li et al., 2010; Samavarchi-Tehrani et al., 2010), with subsequent reversal to a self-renewal and pluripotency state (Silva et al., 2008; Nichols and Smith, 2009; Gillich et al., 2013). Thus, I propose that i-CSCs would be equivalent to ESCs, with one important difference: while an ESC has an integral genic program, an i-CSC would have a program impaired in terminal genome homeostasis (Katoh, 2007; Silva et al., 2008). In this model, an i-CSC would be a p-ESC (Figure 3). At a molecular level, iCSC genesis would presumably require the following steps in the original cell: (a) De-programming (de-differentiation), by impairment of the systems of genomic homeostasis, carried out by growth modulators (p16, p21, p53, p27, E2A, pRb) (Prabhu et al., 1997; Ouyang et al., 2002; Menendez et al., 2010; Tapia and Schöler, 2010) and/or by specific autocrine and paracrine signal pathways (WNT, BMP, LIF, FGF, ERK, TGFb) (Sato et al., 2004; Katoh, 2007; Scheel et al., 2011; Chen et al., 2015), with a consequent differentiation loss and reversal to a previous more primitive (mesenchymal/epithelial/undifferentiated) state (Silva et al., 2008; Schwitalla et al., 2013). (b) Stable PGRN reactivation, by de-regulation of the activity of defined genes (NANOG, OCT4, SOX2, STAT3, ID), with reacquisition of self-renewal and pluripotency (Silva et al., 2008; Do et al., 2013; Yin et al., 2015). (c) Reprogramming, through ID1 gene expression, that prevents i-CSC/CSC0s (p-ESCs) and their direct progeny from normally differentiating (“blocking event”) (Ying et al., 2003; Wang et al., 2012; Nair et al., 2014). ID proteins associate with ubiquitous E proteins, preventing their DNA binding and differentiation activity (Ruzinova and Benezra, 2003; Wang and Baker, 2015). ID1 constitutive expression determines neo-angiogenesis, survival, anti-apoptosis and invasion/migration, both in cancer and embryo development.

Thus, I suggest an iCSC/p-ESC model for the tumor initiation.

### Primary Cancer Stem Cells (CSC1s) as Epiblast Cells

Once generated, i-CSC/CSC0s would survive and symmetrically proliferate early in an anchorage-independent way. Due to its presumed nature as a p-ESC, an i-CSC/CSC0, like an ESC, could generate all cell lineages of the body and, therefore, an initial structure similar to a pre-implantation blastocyst *in vivo* (Figure 1B), or a tumorsphere *in vitro* (Figure 3; Johnson et al., 2013), that is able to implant in a surrounding microenvironment (niche) (Stewart et al., 1992), where ID1 proteins would synchronize stemness and anchorage to the niche (Niola et al., 2012). Here, the p-ESC direct progeny would proliferate in an anchorage-dependent way, with an autocrine symmetric mechanism, generating primary cancer stem cells (CSC1s), presumably corresponding to epiblast cells (Figure 1C). CSC1s would form a “tumor germinal center” that continuously feeds the neo-forming tumor (Figure 2, 3). CSC1s would be blocked in a pluripotent undifferentiated/epithelial state (p-ESCs/p-Epi-SCs) (Nichols and Smith, 2009; Gillich et al., 2013), owing to NANOG, OCT4, SOX2, and STAT3 (PGRN) overexpression (Wen et al., 2010; Miyanari and Torres-Padilla, 2012), or to other genomic conditions inducing ID1 constitutive expression (Ruzinova and Benezra, 2003; Ying et al., 2003; Nair et al., 2014). CSC1s would express self-renewal and pluripotency markers (ALDH1, CD44, CD133) (Wakamatsu et al., 2012). The number of CSC1s would be low, stable and strictly controlled by the “niche contact”, as a limiting factor. ALDH1^+^ CD44^+^ Ki67^+^ CSCs, detected in a central position in mammary tumors, might be hypothetical CSC1s, constituting about 0.084% of the tumor mass (Liu et al., 2014). CSC1s would be TICs, like the identified small population of “slow-cycling melanoma cells,” essential for continuous tumor growth (Roesch et al., 2010), or the “long-term renewing” TICs (LT-TICs), that drive tumor feed and metastasis formation in colon cancer (Dieter et al., 2011).

Thus, I propose that tumor initiation and growth might be continuously sustained by cells (CSC1s) with features (pluripotency and self-renewal) typical of epiblast cells.

### Secondary Cancer Stem Cells (CSC2s) as Hypoblast Cells

When niche contact becomes limiting, only one CSC1 daughter could retain maternal place and phenotype, while the other could acquire a new epithelial phenotype (CSC2) (Ezashi et al., 2005; Quail et al., 2012). CSC2s would correspond to hypoblast cells (Figure 1C) and therefore they would not have self-renewal and pluripotency, differently to CSC1s. However, CSC2s might, eventually, revert to CSC1s when a niche contact becomes available (Ezashi et al., 2005; Quail et al., 2012). The CSC2 phenotype could occur via the LIF-STAT3-RAS-MAPK-ERK-MYC-ID2 pathway, by NANOG inhibition (Kunath et al., 2007; Yamanaka et al., 2010), ID1/2 switching, loss of stemness/pluripotency (Itahana et al., 2003; Park et al., 2013) and acquisition of fast paracrine growth (Iavarone et al., 1994). Hypothetical CSC2s might be the CSCs with an ALDH1^+^ CD44^-^ Ki67^+^ profile detected in mammary tumors (Liu et al., 2014). The “tumor transient-amplifying cells” (T-TACs) with limited or no self-renewal in human colon cancer, and the “rapidly proliferating main population” surrounding the previously mentioned “slow-cycling melanoma cells” (Roesch et al., 2010; Dieter et al., 2011) also could be hypothetical CSC2s. The CSC2 number would be related to the niche volume, nutriments and oxygen quantities (Ezashi et al., 2005; Mohyeldin et al., 2010; Quail et al., 2012): in mammary carcinomas, ALDH1^+^ CD44^-^ Ki67^+^ CSCs (CSC2s) constitute about 5.54% of the tumor mass and lie in a sub-central site, physically distinct from ALDH1^+^ CD44^+^ Ki67^+^ CSCs (CSC1s) (Liu et al., 2014). CSC1s and CSC2s, together, would form a presumable “tumor growth nucleus” responsible for tumor expansion (Figure 3).

Thus, I think that tumor growth might result from an autocrine/paracrine signaling-driven proliferation of cells (CSC2s) with features typical of hypoblast cells.

### Tertiary Cancer Stem Cells (CSC3s) as Mesoderm Precursor Cells

When the niche microenvironment becomes limiting for growth, certain stereotrophic factors, such as hypoxia, and particular autocrine or paracrine signals (LIF, STAT3, TGFb, WNT, NOTCH) (Ezashi et al., 2005; Mohyeldin et al., 2010; Scheel et al., 2011) could induce CSC1s/CSC2s to generate a new phenotype (CSC3), that is able to migrate, invade and search for more favorable survival conditions elsewhere (Sahlgren et al., 2008; Conley et al., 2012; Tiwari et al., 2012). This phenotype would have mesenchymal features, that would result as a downstream effect of the STAT3-RAS-MAPK-ERK-MYC pathway, regulating ID3/E47 interactions and promoting tumor cell migration and invasion (Bain et al., 2001) through expression of mesenchymal genes such as MMPs, SNAIL1, TWIST1, and PRRX1. The CSC3 phenotype would be the result of an EMT that recalls the EMT of mesoderm precursors at the PS (Figure 1D,E). STAT3-SNAIL1 would confer delamination, migration and invasive properties to CSC3s, as TMCs (Barrallo-Gimeno and Nieto, 2005; Thiery et al., 2009; Kamran et al., 2013; Yin et al., 2015). TWIST1 would increase their invasive properties and, together with PRRX1, would favor installation as micro-metastases in metastatic niches (Yang et al., 2008; Eckert et al., 2011; Tran et al., 2011; Ocaña et al., 2012), thus presumably mimicking the onset of somitogenesis at gastrulation sites. ALDH1^-^ CD44^+^ Ki67^-^ CSCs, shown within mammary tumors and located in a peripheral position, at the invasive front, would be presumable CSC3s, constituting about 12.87% of the tumor mass (Liu et al., 2014). CSC1s, CSC2s, and CSC3s, together, would form a “tumor progression module” (Figure 2, 3). I hypothesize that, here, CSC3s with more favorable stereotrophic conditions (normoxia) would become TWIST1^+^ CSC3s, that is able to install and proliferate asymmetrically via ID3/E47, generating more differentiated oligopotent cancer progenitor cells (CPCs). CPCs, in turn, would yield further differentiated cancer cells (CDCs) (Ezashi et al., 2005; Cakouros et al., 2010; Mohyeldin et al., 2010). The resulting CSC1s/CSC2s/CSC3s/CPCs/CDCs, together, would mimic an ectopic, rudimentary somito-histo-organogenesis process (Figure 1F), and would account for the cell hierarchic heterogeneity in tumor progression (Figure 2), where CSCs would be surrounded and protected by CPCs and CDCs (Marjanovic et al., 2013; Liu et al., 2014; Cabrera et al., 2015; Figure 3). On the contrary, CSC3s with less favorable stereotrophic conditions (hypoxia) would undergo EMT, becoming SNAIL1^+^ CSC3s, that would be induced to delaminate and migrate as quiescent circulating micro-metastases (Ezashi et al., 2005; Sahlgren et al., 2008; Mohyeldin et al., 2010; Conley et al., 2012; Tiwari et al., 2012; Figure 3).

Thus, I propose that tumor progression might occur by appearance of cells (CSC3s) with features of mesoderm precursor cells, that are able to migrate, invade and colonize new sites.

### Micro-Metastases and Macro-Metastases as Localization, Induction, and Growth of Gastrula Migrating Cells

After installation as micro-metastases in defined metastatic sites (Arvelo et al., 2016), by specific niche signals, SNAIL1^+^ CSC3s would undergo MET (Chaffer et al., 2006; Brabletz, 2012; Ocaña et al., 2012; Teo et al., 2014), reverting to self-renewing CSC1s (TICs), that are able to finally generate macro-metastases (Figure 2, 3). Development of macro-metastases would occur via a hypothetical bidirectional genic system TWIST1/ID3/E47/ID1/PRRX1/CD44/PGRN, balanced by TGFb (Gupta et al., 2007; Bourguignon et al., 2012; Ocaña et al., 2012; Stankic et al., 2013; Teo et al., 2014; Wang and Baker, 2015). This system would form a genic switch point in (re)programming: back, for stemness (MET) via PRRX1-CD44-PGRN-ID1 and, forward, for growth-differentiation via ID3-E47-TWIST1 (Stankic et al., 2013). This would lead, in macro-metastases, simultaneously to self-renewal, angiogenesis and differentiation growth, like in the primary tumor. Presumably, PRRX1 downregulation would suppress the mesenchymal state, restoring the epithelial state (MET) through PGRN reactivation via CD44-ID1. It has been shown that downregulation of PRRX1 expression is necessary for micro-metastases to undergo MET and generate macro-metastases (Brabletz, 2012; Ocaña et al., 2012; Hirata et al., 2015), conferring TIC properties to quiescent CSC3s via an ID3/E47/ID1 switch (O’Brien et al., 2012; Hirata et al., 2015; Wang and Baker, 2015; Figure 3). ID1 and ID3 are necessary for TIC functions in the genesis of both primary tumors and metastases, sustaining proliferation in early stages via p21 (Gupta et al., 2007; O’Brien et al., 2012). Moreover, ID1 and ID3 are required for angiogenesis and vascularization of tumor xenografts (Lyden et al., 1999), necessary for macro-metastasis development. TGFb/ID1 signals promote metastatic colonization via a MET, antagonizing TWIST1 EMT (Gupta et al., 2007; Stankic et al., 2013) in normoxic metastatic sites, but not in hypoxic primary tumor sites (Mohyeldin et al., 2010), where EMT is governed by SNAIL1 (Stankic et al., 2013). I hypothesize that EMT/MET switching could occur through ID3/E47/ID1 balance (Bain et al., 2001; Cubilio et al., 2013; Bohrer et al., 2015; Wang and Baker, 2015) and that it could involve ID3b and ID1b isoforms, generated by alternative splicing of the ID1 and ID3 genes (Deed et al., 1996; Tamura et al., 1998).

## Crucial Molecular Factors Common to Embryos and Cancer

### Pre-implantation Embryos/Initial Tumors (Initiation)

Pre-implantation embryos lie in unfavorable nutriment and microenvironment conditions. Thus, it is necessary they reach and are installed in a niche that is able to supply proper stereotrophic factors for survival. To this end, molecules such as HSP70, HLA-G, ID, and LIF are crucial both for embryos and cancer.

ID Proteins - ID1, ID2, ID3, and ID4 proteins are highly expressed in normal ontogenetic development, where their function is associated with the primitive proliferative phenotype, and regulate differentiation associating to ubiquitous E proteins (Ruzinova and Benezra, 2003; Wang and Baker, 2015). Numerous studies indicate that ID1, ID2, and ID3 have an oncogenic function, whereas ID4 promotes the survival of adult SCs, differentiation and/or differentiation time (Patel et al., 2015). ID proteins are key regulators of CSCs and tumor aggressiveness (Lasorella et al., 2014). During distinct stages of breast metastases, ID proteins mediate phenotypic switching of CSCs (Stankic et al., 2013) and control CSC niches in an autocrine/paracrine way (Niola et al., 2012; Nair et al., 2014). ID1 has multiple roles in cancer progression, such as implantation in primary and metastatic niches, angiogenesis, CSC survival, chemoresistance, growth, apoptosis inhibition and activation of WNT signaling (Ling et al., 2006; Niola et al., 2012; Nair et al., 2014). In particular, the ID1b isoform has been shown to maintain cell quiescence, confer self-renewal and CSC-like properties, and impair malignancy, inhibiting proliferation and angiogenesis (Tamura et al., 1998; Nguewa et al., 2014; Manrique et al., 2015). Thus, ID1b could be related to slow symmetrical self-renewal, niche anchorage and pluripotency maintenance. The ID1a isoform, on the contrary, could be related to fast asymmetrical self-renewal, angiogenesis and evolutionary growth (Lyden et al., 1999), allowing ID1/ID2 switching, with overcoming of pluripotency and subsequent lineage commitment, like in epi/hypoblast segregation. ID2 enhances cell proliferation by binding pRb, an inhibitor of cell cycle progression, and is directly repressed by p53 (Iavarone et al., 1994; Paolella et al., 2011). A WNT-bCAT signal increases ID2 expression level and the incidence of CSC-like phenotype, mediating the effects of hypoxia on the breast and colorectal CSC hierarchy (Rockman et al., 2001; Dong et al., 2016). I hypothesize that ID2 would be involved in overcoming stemness/pluripotency, thus leading to cell commitment and fast autocrine/paracrine growth, via LIF-STAT3-RAS-MAPK-ERK-MYC (Park et al., 2003; Pankaj et al., 2013), with ERK blocking NANOG-ID1 (Itahana et al., 2003; Kunath et al., 2007; Park et al., 2013). ID1/ID2 switching would occur via ID1-WNT/bCAT-ID2, induced by hypoxia (Rockman et al., 2001), and might be related to the epi/hypoblast and CSC1/CSC2 transitions. Moreover, ID2 expression might lead, via RAS-MAPK-ERK, to the activation of ID3 genes (Bain et al., 2001), with subsequent ID2/ID3 switching and mesenchymal gene expression, namely to EMT. Because the RAS-MAPK-ERK cascade regulates ID3/E2A (E47) interaction (Bain et al., 2001), and ID3/E47 balance would determine stem/precursor differentiation (Bohrer et al., 2015), and because ID3 interacts with ID1 (Wang and Baker, 2015), I hypothesize that ID3/E47/ID1 balance would be related to the potential stemness of mesenchymal phenotypes, such as MSCs and CSC3s (O’Brien et al., 2012; Cubilio et al., 2013; Teo et al., 2014; Bohrer et al., 2015). In more detail, the ID3a isoform could be related to overcoming of the CSC3 SNAIL1^+^ mesenchymal state and to the stem/progenitor switch via TWIST1 (Cakouros et al., 2010; Tran et al., 2011; Bohrer et al., 2015). ID3b isoforms, on the contrary, would be related to the maintenance of the SNAIL1^+^ mesenchymal state of dormant CSC3s and, thus, to a potential EMT/MET switch for reversion to the CSC1 (TIC) phenotype. Indeed, ID3b inhibits vascular formations (Deed et al., 1996; Forrest et al., 2004) and, thus, the possibility of macro-metastasis development, maintaining dormancy. ID4 acts as an ID1/ID2/ID3 inhibitor and promotes E47 binding and transcriptional activity in a differentiation direction (Umetani et al., 2004; Yun et al., 2004; Sharma et al., 2015). ID4 is a potential tumor suppressor (Umetani et al., 2004; Carey et al., 2009) and suppresses MMP2-mediated invasion of glioblastoma-derived cells by direct inactivation of TWIST1 (Rahme and Israel, 2015). I hypothesize that ID4 might be related to terminal differentiation growth, via E47, and to global genomic homeostasis (Umetani et al., 2004; Sharma et al., 2015). Thus, ID proteins seem to be crucial factors both in cancer and embryos.

*HSP70* (heat shock protein 70 kDa) molecules are precociously expressed in ontogenesis, from zygotic gene activation, through blastulation, implantation, gastrulation, and organogenesis to fetal maturation (Figure 1A–G) (Bensaude et al., 1983; Luft and Dix, 1999). In early ontogenesis and oncogenesis, HSP70 could be a first system of protection and survival for pre-implantation embryos, as well as for initial tumors. Protective action of HSP70 for pre-implantation embryos might occur at an endocellular level for preventing apoptosis (Samali and Cotter, 1996); in effect, HSP70 knockdown renders embryonic cells weaker and apoptotic (Neuer et al., 1998). A small amount of HSP70.1-3 is necessary for pre-implantation embryogenesis (Luft and Dix, 1999). HSP70 pre-implantation expression includes a constitutive component (HSC70 and HSP70.1-3) from zygote to four-blastomer morula, and a component (HSP70.1-2) inducible by heat and chemical agents in four- to eight-blastomer morula and in blastocyst (Figure 1A,B) (Luft and Dix, 1999). In adult normal tissues, HSP70s are normally absent, except for transient expression in normal mitosis (Stangl et al., 2011).

HSP70 deregulated overexpression is associated with tumor transformation. In human tumor cells, global profiling of the surface proteome has revealed HSP70.1-2 abundance (Stangl et al., 2011). HSP70s are highly expressed in various tumor cell types, thus rendered resistant to adverse microenvironments and chemotherapy (Shu and Huang, 2008). In human melanoma cell lines, HSP70.1-2 constitutive expression occurs (Dressel et al., 1998). During tumor development, HSP70s can be expressed on the cell surface or exported in the circulation (Shu and Huang, 2008). Human tumor cell lines of colon, breast, lungs and melanomas bind anti-HSP70.1 monoclonal antibodies (mAbs) (Stangl et al., 2011); highly metastatic tumors, but not their primary or poorly malignant counterparts, express membrane HSP70 (mHSP70) (Stangl et al., 2011). mHSP70^+^ tumors actively release lipidic vesicles (exosomes) with an HSP70^+^ surface (Stangl et al., 2011). Therefore, I suggest that HSP70s might constitute a first important system of protection and survival in early ontogenesis and oncogenesis.

*HLA-G* (human leukocyte antigen-G) molecules could be a second important system of protection and survival both for initial embryos and cancer. HLA-Gs appear to be evolutionally and genetically linked to HSP70s: heat shock, a major inductor of HSP70.1-2 expression, and arsenite chemical shock also induce HLA-G expression in tumor cell lines (Ibrahim et al., 2000; Yao et al., 2005). In human pre-implantation embryos, HLA-Gs are expressed as several isoforms, including HLA-G1 and HLA-G5. HLA-G1s are already expressed in two- to eight-blastomer embryos and in all blastocysts as membrane-bound molecules (mHLA-G), whereas, HLA-G5s are expressed only from the blastocyst onwards, as soluble forms (sHLA-G) secreted in biological liquids, or generated by mHLA-G shedding (Yao et al., 2005). HLA-Gs circulating in biological fluids might also be associated with extracellular vesicles (EVs) (Rebmann et al., 2016), containing too antigens, ligands, receptors, cytokines, GFs, mRNAs, and miRNAs (da Silva Nardi et al., 2016). Human pre-implantation embryos express and secrete HLA-Gs, the level of which might be predictive of their implantation capacity (Yao et al., 2005). HLA-Gs orchestrate the early interaction of human trophoblasts with the maternal niche (Gregori et al., 2015). HLA-Gs are expressed early in human ICM and ESCs but, later, mainly in invasive TE, and no more in ICM; after implantation, HLA-Gs are expressed in hypoblast, but no longer in epiblast; yolk-sac mesoderm, endothelial cells of developing vessels, mesenchymal cells and progenitor cells express sHLA-Gs (Hunt et al., 2005; Yao et al., 2005; Verloes et al., 2011) (Figure 1). During pregnancy, sHLA-Gs can be detected in maternal serum. In adult normal tissues, HLA-G constitutive expression is mainly restricted to the fetal extravillous trophoblasts (EVTs), which invade the maternal decidua, rich in NK cells and macrophages (Tilburgs et al., 2015).

Tumor and mesenchymal cells also secrete HLA-Gs in EVs (Yen et al., 2009; Burrello et al., 2016). Tumor cells utilize EVs for dictating a defined phenotype to surrounding cells (Naito et al., 2017). Recent data show that tumor EVs contain molecules for intercellular communications (da Silva Nardi et al., 2016; Rebmann et al., 2016), that act on and impair the recipient immune cells, favoring immune evasion, initiation, tumor development, angiogenesis, invasion, metastasis, CSC and EMT preservation and chemoresistance (Sheu and Shih, 2010; Kosaka, 2016). HLA-G expression has been shown in 22/33 primary tumor tissues of human ovarian carcinoma, but not in normal tissue (Lin et al., 2007), and in 30% of surgically removed melanoma lesions (Yan et al., 2005). In lung cancer, HLA-G1/5 upregulation associates with a high-grade histology, HLA-Ia loss and immunosuppressive IL10 production (Urosevic et al., 2001). Increased HLA-G expression correlates with immune evasion during colorectal cancer progression (Fukushima et al., 1998) and in gastric cancer (Du et al., 2011). These data might, thus, indicate that, in general, in embryos and cancer, HLA-Gs and HSP70s might act not only as protective shields in potentially adverse environments (Sheu and Shih, 2010), but also as means for their invasion and colonization (Yao et al., 2005). More in detail, it seems that HLA-G1 is preferentially expressed precociously and in more peripheral embryonic structures in an essentially protective scope, whereas HLA-G5 is produced later and in invasive embryonic structures and in tumor metastatic cells, in order to favor protection, invasion, installation and development in the host (Rouas-Freiss et al., 2007; Sheu and Shih, 2010). HLA-Gs are induced by hypoxia via HIF-1a (Rebmann et al., 2003) and are upregulated by IL10 with autocatalytic feedback (Urosevic et al., 2001, 2002; Stankic et al., 2013). HLA-Gs induce IL6 production (Urosevic et al., 2002), and thus activation of the gp130-STAT3 pathway, regulating proliferation, invasion, migration and angiogenesis (Kamran et al., 2013). In my opinion, all these data suggest that HLA-G molecules could be key inter-players and shared actors in the initiation and maintenance of both embryogenesis and carcinogenesis. Particularly, HLG together to HSP70, would represent an essential system for protection and survival of both embryo and tumor.

### Peri-Implantation Embryos/Primary Tumors (Implantation and Growth)

Beyond a certain stage, a blastocyst might develop further only if a supply of nutrients is possible from the outer environment: this would implicate for the embryo the necessity of implantation in the maternal uterus, and for initial tumors in a niche. Blastocyst implantation occurs thanks to the structure and properties of the syncytiotrophoblast (Figure 1C,D; Park et al., 2003) and to a parallel maternal endometrium condition, suited to accepting implantation. The process requires some crucial interacting factors, such as LIF/LIFr, IL6/IL6r, IL10, IL11, GP130, JAK, and STAT3, and correlated signal pathways, leading to the switch from anchorage-independent to anchorage-dependent growth, both in embryos and cancer.

LIF/LIF-r, IL6/IL6-r, gp130-JAK-STAT3 - In peri-implantation, LIF (leukemia inhibitory factor) is crucial: in LIF knockout mice, blastocyst implantation does not occur; in women, LIF strongly increases in the implantation window (Sherwin et al., 2002; Aghajanova, 2004). Uterine expression of LIF coincides with the onset of blastocyst implantation, and this depends on maternal expression of LIF (Bhatt et al., 1991; Cheng et al., 2001; Sherwin et al., 2002; Yue et al., 2015). LIF is essential for inducing a receptive uterus, but not for embryogenesis (Chen et al., 2000). LIF and LIFr expression in the human endometrium suggests a potential autocrine/paracrine function in regulating embryo implantation (Cullinam et al., 1996). In implantation, LIF carries on its biological functions mainly by activation and regulation of the JAK-STAT3, AKT, ERK1-2, and MAPK signal pathways, inducing expression of integrin α5β1, that realizes implantation, endothelial proliferation and endometrial vascularization (Cheng et al., 2001; Sherwin et al., 2002; Park et al., 2003). LIF-gp130-STAT3 is also linked to HLA-G expression through IL6 and IL10 (Urosevic and Dummer, 2003; Yue et al., 2015). In initial embryos, HLA-G expression is a fundamental prerequisite for obtaining pregnancy: indeed, in *in vitro*-fertilized human embryos, only those whose culture surnatant contains sHLA-G are able to perform implantation (Fuzzi et al., 2002; Alizadeh et al., 2016).

A LIF signal is also expressed at high levels in a wide spectrum of human cancers, including melanomas, skin, kidney, prostate, pancreas and breast cancer, where cell proliferation is stimulated by paracrine and autocrine pathways, as in embryo implantation (Cullinam et al., 1996; Kellokumpu-Lehtinen et al., 1996). The amount of LIF secreted by a tumor seems to regulate cancerogenesis (Guo et al., 2015); LIF overexpression in breast cancer patients is significantly associated with an unfavorable rate of survival without relapses (Guo et al., 2015). High expression of LIFr identifies very malignant melanocytic lesions at an early stage, and it is a crucial condition for the nevus/implanted melanoma transition (Guo et al., 2015). Analysis of 441 melanomas and 90 nevi showed low LIFr expression for all nevus stages, whereas the presence of this receptor starts to increase in dysplastic nevi, with significantly higher expression in primary melanomas (implantation), and even higher in metastatic melanomas (Guo et al., 2015), suggesting a striking correlation between LIF/LIFr expression and oncogenesis. LIFr knockdown inhibits melanoma cell migration in wound-healing tests (Guo et al., 2015). In general, LIFr activation can promote metastasis and increase the invasion potential of solid tumors (Guo et al., 2015). In human colorectal cancer cells and in solid tumors, hypoxia is an important factor inducing LIF mRNA expression, mediated mainly by HIF-2a (Yue et al., 2015). TGFb also induces expression of LIF mRNA; LIF induction is important to maintain the self-renewal of glioma-initiating cells and prevent their differentiation (Yue et al., 2015). LIF binds to human breast cancer cells and stimulates their proliferation (Estrov et al., 1999). I hypothesize that in the tumor process, LIF/LIFr might be at the basis of: (a) implantation of the primary tumor (CSC1s) via gp130-JAK-STAT3-ID1, with self-renewal and pluripotency (Niola et al., 2012); (b) subsequent paracrine growth (CSC1s/CSC2s) via gp130-JAK-STAT3-RAS-MAPK-ERK-MYC-WNT-ID2 (Cheng et al., 2001; Park et al., 2003); (c) genesis of cells with a mesenchymal phenotype (CSC3s) via CD44-STAT3-RAS-SNAIL1-MMPs-ID3-TWIST1 (Bain et al., 2001; Cheng et al., 2006; Su et al., 2011; Zeilstra et al., 2014; Yan et al., 2015). All these data clearly indicate that LIF/LIFr and related signaling pathways are crucial in cancer initiation, implantation, growth and diffusion, in a way that recalls their function in embryo development.

### Post-implantation Embryos/Metastatic Tumors (Progression)

In the post-implantation embryo, at the PS, cells with a mesenchymal phenotype appear, migrating between epiblast and hypoblast cells (Figure 1D). The mesenchymal phenotype also characterizes metastatic TMCs (Sahlgren et al., 2008; Thiery et al., 2009; Ocaña et al., 2012). The main factors that correlate with the embryonic mesenchymal state, tumor metastases and progression include HLA-G, HSP70, hypoxia, STAT3, CD44, SNAIL1, TWIST1, PRRX1, TGFb, WNT/bCAT, ID, and MMPs. Mesenchymal progenitors derived from hESCs (EMPs) express surface HLA-G1 (Yen et al., 2009). Mesenchymal cells and progenitor cells also express HLA-Gs. IL10 selectively induces HLA-G expression in human invasive trophoblasts and monocytes (Moreau et al., 1999). In mesenchymal CSCs of kidney cancer, HLA-Gs and EVs enhance metastasis and progression (Grange et al., 2015). Highly metastatic tumors show the presence of membrane HSP70s (Stangl et al., 2011). In progression of many cancer types, migration is stimulated by the LIF-STAT3 pathway, constitutively activated, that regulates proliferation, migration, angiogenesis and metastasis (Kamran et al., 2013; Guo et al., 2015). The mesenchymal phenotype is highly characterized by the surface marker CD44.

*CD44* is a cell-surface glycoprotein constituting a signal platform that regulates the expression of genes related to cell-matrix adhesion, migration, proliferation, survival and differentiation in development (Okamoto et al., 1999; Williams et al., 2013; Yan et al., 2015). The main CD44 receptor is hyaluronic acid (HA), a component of the extracellular matrix (ECM), that envelops tumor cells and bulk, regulating proliferation and motility in cancer progression and metastasis (Chanmee et al., 2015; Avnet and Cortini, 2016). The CD44-STAT3 complex induces Cyclin D1, MMP9, HIF-a2, c-MYC, TWIST1, cytoskeleton remodeling (Su et al., 2011; Yan et al., 2015) and activation of RAS signaling (Cheng et al., 2006). Expression of CD44s (standard) is ubiquitous, while CD44v (variant) isoforms seem restricted to aggressive tumors (Zeilstra et al., 2014). CD44s/CD44v switching is a critical event during EMT (Yan et al., 2015).

CD44v (v3, v6, v8) isoforms are CSC markers and have a crucial role in regulation of stemness, self-renewal, tumor initiation, metastasis and chemoresistance (Bourguignon et al., 2012; Chanmee et al., 2015; Yan et al., 2015; Avnet and Cortini, 2016). A positive feedback loop couples RAS activation and CD44v isoform expression (Cheng et al., 2006). CD44/osteopontin (the main component of metastatic niches) interaction activates NANOG-STAT3, OCT4-SOX2-NANOG and c-MYC, namely PGRN, and it is induced by hypoxia via HIF-a1 (Krishnamachary et al., 2012; Pietras et al., 2014). CD44 transcription and cell growth are suppressed by p53 (Godar et al., 2008). Thus, I hypothesize that CD44v could be a sort of molecular trigger that is able to directly activate PGRN in the initial MET for reprogramming (Su et al., 2011; Yan et al., 2015) and, thus, a crucial factor in the generation of signaling for the switch between mesenchymal and epithelial states, usually occurring both in embryos and cancer.

## Immunological Aspects Common to Embryos and Cancer: Host Tolerance and Favoring

HSP70s and HLA-Gs are able to strongly influence many important functions of the immune system. Membrane HSP70s affect NK cell cytotoxicity by acting as recognition/activator ligands (Luft and Dix, 1999; Stangl et al., 2008), whereas HLA-Gs are able to inhibit practically all immune system components (NK, T, DC, and B cells) (Hofmeister and Weiss, 2003; Hunt et al., 2005; Gros et al., 2008; Loumagne et al., 2014; Gregori et al., 2015; Tilburgs et al., 2015; Lopatina et al., 2016). In ESCs and pre-implantation embryos, mHLA-G and sHLA-G block all the uterine immune cells, binding the KIR2DL4 inhibitory receptors on NK cells, and ILT2 (LILRB1) and ILT4 (LILRB2) inhibitor receptors on all the leukocytes and macrophages (Hofmeister and Weiss, 2003; Li et al., 2004; Verloes et al., 2011; Tilburgs et al., 2015; Rebmann et al., 2016). Both membrane and soluble HLA-Gs would confer immune-suppressive properties to the cells producing them, in various ways: (a) For HLA-G1, direct interaction with ILT2 and ILT4, determining CD8^+^ T cell apoptosis, NK cell immobilization, and impairment of monocyte/DC differentiation, with a high production of IL4 and IL10 immunosuppressive cytokines (Rebmann et al., 2003; Urosevic and Dummer, 2003; Gros et al., 2008; Gregori et al., 2015; Rebmann et al., 2016). Mesenchymal progenitors derived from hESCs (EMPs) strongly suppress NK and T cells by HLA-G1 (Yen et al., 2009). (b) For induction of cascade signaling and biological activity in the host cells, thanks to the factors contained in EVs, internalized by trogocytosis or pinocytosis (Rouas-Freiss et al., 2007). CSCs cross-talk with MSCs through EVs containing mRNA and miRNA, identified as the main factors responsible for the phenotypic changes induced in the cells receiving EVs (Lopatina et al., 2016). (c) For amplification of mechanisms and conditions favorable to the cells releasing EVs, with further involvement of other surrounding cells. In human neuroblastomas, tumor cells instruct monocytes to produce and release sHLA-G (Rouas-Freiss et al., 2003). HLA-G transfer from antigen-presenting cells (APCs) to activated T cells has been reported in embryos, with reversion of these cells to a regulator phenotype blocking the allo-immune response (Rebmann et al., 2016). A co-culture of activated decidual NK (dNK) cells or peripheral NK (pNK) cells with EVTs results in mHLA-G initial acquisition within 18 h, and complete acquisition in 36 h (Tilburgs et al., 2015).

In tumors, HLA-Gs transfer quickly from APCs or cancer cells to T and NK cells, and convert these cells into temporarily suppressor HLA-G^+^ cells (Rebmann et al., 2003). Once acquired by NK cells, HLA-Gs are degraded, and pNK cells revert to their previous cytotoxic phenotype (Tilburgs et al., 2015). Both HLA-G5 and HLA-G1 endocytosed by NK cells lead to NFkB pathway activation and, finally, to transcription of immunosuppressor (IL10) and pro-angiogenetic (IL6) factors (Rebmann et al., 2016). It has been shown that pNK cells might acquire via trogocytosis HLA-Gs from a transfected melanoma M8 cell line, and that, after acquisition, NK cells are no longer cytotoxic and are unable to realize immune synapses (Lesport et al., 2009; Tilburgs et al., 2015). In gastric cancer, HLA-G overexpression associates with immune escape and correlates with a local increase of T regulatory cells (TREGs) (Du et al., 2011). NK cytolysis depends on the amount of HLA-G1 expressed, that in malignant tumors can go from 0 to 100%, with complete NK inhibition, reduced local and systemic immunosurveillance and tumor progression (Rebmann et al., 2003; Chen et al., 2013). HLA-G1s and HLA-G5s have an additive suppressor effect on NK cytolysis dependent on their level, but HLA-G5 is a more potent inhibitor (Zhang et al., 2014). Within a population of HLA-G^-^ tumor cells, few HLA-G^+^ cells have significant immune inhibitory effects (Lesport et al., 2009). HLA-G upregulation also occurs through factors such as cytokines (IL10), stress and chemotherapeutic demethylating agents (Rouas-Freiss et al., 2003; Yan et al., 2005). IL10 upregulates HLA-G, that induces an immunosuppressive Th2 profile (Urosevic et al., 2002; Rebmann et al., 2003), with further IL10 increase in a vicious circle (Moreau et al., 1999). Interaction of HLA-G^+^ cells with NK cells also enhances IL6 production, that induces angiogenesis and SC proliferation via gp130-JAK-STAT3-OCT4. The inhibiting effects of HLA-G on NK cells are eliminated by the action of IL15, IL2 and IL12 (Tilburgs et al., 2015). CSC immune evasion also occurs through shedding of MIC-A/B, HSP70 and HLA-G1 (Ames et al., 2015). It has been shown that highly metastatic tumors, but not their primary or poorly malignant counterparts, are mHSP70^+^, capable of shedding (Stangl et al., 2011). It is presumable that a high number of molecules such as HSP70, HLA-G, and MIC-A/B released in circulation could lead to ectopic activation and subsequent neutralization of immune cells, without these coming into contact with CSC targets (Stangl et al., 2011), thus favoring diffusion of metastasis.

## Current Anticancer Therapeutic Approaches in Agreement With the I-CSC/P-ESC Model

Several current partially successful approaches for cancer therapy are in agreement with the i-CSC/p-ESC model, targeting crucial factors common to embryos and tumors, such as HSP70, HLA-G, ID, LIF/LIFr, and CD44. CSCs seem to be preferential targets for NK cells through upregulation of antigens (HSP70, MIC-A/B, FAS, DR5) induced by stress, by which NK cells are capable of targeting (only) quiescent, non-proliferating cells (Ames et al., 2015). Thus, in solid tumors, slow-renewing or quiescent CSCs might be more easily killed by NK cells after depletion of proliferating non-CSCs (CPCs) through anti-proliferative therapies (Ames et al., 2015).

HLA-Gs are a valid target in cancer therapy (Lin and Yan, 2015; Zhang et al., 2018); cytotoxicity studies show that HLA-Gs drastically inhibit lysis of human ovarian carcinoma cells, with subsequent immune evasion, and that lysis can be restored by the conformational anti-HLA-G mAb 87G (Tilburgs et al., 2015). Interestingly, dNK cells in culture with IL2, IL12, and IL15 lose the acquired surface HLA-Gs by internalization and degradation, reacquiring cytolytic activity (Tilburgs et al., 2015). In breast cancer patients treated with NACT (neo-adjuvant chemotherapy), high levels of HLA-Gs/EVs before NACT correlate to tumor progression and the presence of circulating tumor stem-like cells, releasing EVs, while high sHLA-G levels (presumably from tumor lysis) indicate a better clinical outcome (König et al., 2016). HLA-G expression is induced in the melanoma cell line OCM-1A after treatment with 5-aza-2’-deoxycytidine (Yan et al., 2005).

Anticancer therapeutic approaches based on endocellular and exocellular HSP70s have been carried out (Didelot et al., 2007; Shu and Huang, 2008; Jego et al., 2010). It has been reported that mHSP70^+^ tumors actively release lipid vesicles (exosomes) with an HSP70^+^ surface, and that they can attract already activated NK cells (but not resting NK cells) (Stangl et al., 2011), causing their ectopic degranulation and neutralization. The TKD peptide (14 amino acids common to all HSP70s), combined with IL2 low doses, has been found to stimulate NK cell migratory and cytolytic activity against HSP70^+^ tumor cells (Stangl et al., 2011). A murine antibody anti-TKD (cmHsp70.1 mAb) binds all human vital tumor cell lines of colon, breast, lungs and melanomas; thus, HSP70s could be an immune-therapeutic target in a wide spectrum of tumor types (Shu and Huang, 2008; Stangl et al., 2011). In fact, cmHsp70.1 mAb injected into mice with CT26 colon tumors can significantly reduce the bulk of mHSP70^+^ tumors and increase survival via ADCC induction, that can be further enhanced by NK cells pre-activated with TKD/IL2 (Stangl et al., 2011).

ID genes and proteins are a promising target in cancer therapy for inhibiting tumor cells and their supply in the blood (Benezra, 2001; Fong et al., 2004; Iavarone and Lasorella, 2006). ID protein inhibition by a peptide aptamer induces cell cycle arrest and apoptosis in ovarian cancer cells (Mern et al., 2010a). Suppression of invasion and metastasis in aggressive salivary and breast cancer cells is targeted through inhibition of ID1 expression (Fong et al., 2003; Murase et al., 2016). Inactivation of ID1 genes induces sensitivity of prostate cancer cells to chemotherapeutic drugs (Wong et al., 2008). Co-suppression of ID1 and ID3 results in a significant reduction in the proliferation rate, invasiveness and anchorage-independent growth, reduced angiogenesis and increased apoptosis in small-cell lung cancer (Chen et al., 2014), and significantly reduces the ability of gastric cancer cells to form peritoneal metastases (Tsuchiya et al., 2005). Moreover, ID1 and ID3 knockdown inhibits the metastatic potential of pancreatic cancer, both for proliferation and migration (Shuno et al., 2010). Targeting ID1 and ID3 by a specific peptide aptamer induces E-box promoter activity, cell cycle arrest and apoptosis in breast cancer cells (Mern et al., 2010b). TGFb receptor inhibitors target the CD44^high^/Id1^high^ glioma-initiating cells in human glioblastoma (Anido et al., 2010). It has been shown that ID2 knockdown by an inhibitor of WNT-bCAT signaling markedly suppresses the formation of CSC spheres *in vitro*, and metastases *in vivo* (Rockman et al., 2001), by inhibition of CSC-like phenotypes (Jang et al., 2015).

LIFr expression is a crucial condition for nevus/implanted melanoma transition; LIFr knockdown inhibits migration of melanoma cells in wound-healing tests; thus, LIFr could be a potential target for developing therapies in initial tumor interventions (Guo et al., 2015). Neutralizing antibodies knock down activity or expression of LIF and reduce *in vitro* the stem cell-like properties of murine slow-growing CSCs (American Association for Cancer Research, 2012).

CD44 is a biomarker and therapeutic target in CSCs (Su et al., 2011; Yan et al., 2015), and CD44v is a promising approach for elimination of CSCs (Jin et al., 2006; Orian-Rousseau and Ponta, 2015; Yan et al., 2015). In breast cancer, cells with an EMT phenotype can be inhibited by Abs specific for CD44 (Yan et al., 2015); knockdown of CD44 induces differentiation of breast CSCs and is a promising differentiation therapy (Pham et al., 2011). CD44 targeting reduces tumor growth and prevents post-chemotherapy relapse of human breast cancer xenografts (Marangoni et al., 2009). Inhibition of CD44v3 and v6 by A5G27 peptide copolymer blocks tumor invasion and metastatic colonization (Zaiden et al., 2017). DNA vaccination with CD44v isoforms reduces mammary tumor local growth and lung metastases (Wallach-Dayan et al., 2008).

Immunological approaches to target CSCs have been carried out, and through CSC vaccination, significant antitumor immunity can be conferred (Ning et al., 2012; Pan et al., 2015). For improving the efficacy of breast cancer treatment, a combination therapy has been used, that permits targeting of both CSC-like and bulk tumor cells (Wang et al., 2016). Thus, it is evident that many current therapeutic strategies are addressed to target crucial factors, common to embryos and cancer, at the basis of this i-CSC/p-ESC model.

## Discussion and Conclusion: Proposals for Innovative Cancer Therapy and Prophylaxis Strategies

### Considerations for a Proper Antitumor Therapeutic Strategy

From the numerous experimental data reported above, it is evident that embryo and tumor development occurs in very similar physio-pathological conditions of immune tolerance by the host, which accepts and even favors them. Anticancer therapies carried out so far, targeting separately the factors HLA-G, ID, LIF, HSP70, and CD44, have got important positive, but only partial and non-resolutive, results. This limit might be due to several factors: (a) a particular hierarchic tumor structure, immunologically protective for CSCs, surrounded by CPCs and CDCs (Nishimura et al., 2012; Marjanovic et al., 2013; Liu et al., 2014; Ames et al., 2015; Cabrera et al., 2015); (b) interconversion among CSCs (CSC1/CSC2/CSC3/CSC1) via ID1, ID2 and ID3 proteins (Hermann et al., 2007; Biddle et al., 2011; Dieter et al., 2011; Brabletz, 2012; Quail et al., 2012; Do et al., 2013; Cabrera et al., 2015); (c) the presence of HLA-G and HSP70 on the surface of CSCs and CPCs, with impairment of various immune surveillance mechanisms, NK activity in particular (Samali and Cotter, 1996; Rouas-Freiss et al., 2007; Sheu and Shih, 2010; Stangl et al., 2011); (d) anti-proliferative chemotherapies, that only eliminate actively proliferating CCs (CSC2s and CPCs), but spare slow-renewing CSCs (CSC1s) in niches, or quiescent circulating CSCs (CSC3s), and non-proliferating CDCs (Ames et al., 2015; Wang et al., 2016); (e) release into the circulation of a high quantity of HSP70 by shedding, exosomes or chemotherapeutic necrosis (Shu and Huang, 2008; Stangl et al., 2011), attracting ectopically activated NK cells and neutralizing them at a distance from CSC targets (Stangl et al., 2011); (f) release into the circulation, by shedding, EVs or chemotherapeutic necrosis, of high amounts of HLA-G, that is able to block any immune mechanism, or even instruct normal cells to favor the tumor (Hofmeister and Weiss, 2003; Li et al., 2004; Rouas-Freiss et al., 2007; Gros et al., 2008; Loumagne et al., 2014; Lopatina et al., 2016); (g) negative collateral effects of anti-angiogenetic therapies that, by blocking tumor vascularization, might induce hypoxia and a subsequent increase of metastatic CSCs (CSC3s) (Conley et al., 2012).

### General Suggestions for a Potential Multi-Target Multistep Cancer Therapy

On the basis of the previous considerations, I suggest some general indications for a multistep multi-target therapeutic strategy. To this end, I think that it would be necessary to make use of “micro-antibodies” (mi-Abs), which are artificial, chemically synthetized short chains of amino acids, copied from fully functional natural antibodies (Heap et al., 2005). Their small size allows them to leave the circulation quickly and reach specific target sites in the tissues, normally unapproachable for mAbs. mi-Abs are poorly immunogenic and do not stimulate an immune response versus the host. mi-Abs show neutralizing properties versus viruses, such as HIV, infecting cells *in vitro* (Fujii, 2009). All these mi-Ab properties might consent the following antitumor therapeutic strategy, leading to progressive back-dismantling of the tumor hierarchic organization, selectively targeting the diverse tumor cell populations in a defined multistep sequence: (a) co-culture of autologous peripheral blood mononuclear cells (PBMCs) with IL15, IL12 and IL2, to restore them from the effects of HLA-G (Tilburgs et al., 2015) and dispose of HLA-G-free PBMCs, to be used in next steps; (b) tumor biopsy for the selection of the various populations: differentiated non-proliferating cells (CDCs), non- or slow-proliferating SCs (CSC1s and CSC3s), actively proliferating cells (CSC2s and CPCs); (c) co-culture of HLA-G-free PBMCs with each selected tumor population, and “in toto” tumor populations, previously treated with anti-HLA-G and anti-HSP70 mi-Abs, allowing *in vitro* PBMC sensitization without conditioning; (d) anti-HLA-G and anti-HSP70 prophylaxis of the subject by specific mi-Abs; (e) reinfusion of anti-CDC-sensitized PBMCs and/or chemotherapeutic treatment, to reduce the tumor bulk and expose the internal cell populations (Ames et al., 2015; Wang et al., 2016), thus making them more approachable in the next steps; (f) chemotherapeutic anti-proliferative treatment of the subject to eliminate fast-proliferating CSC2s and CPCs, upon protection with anti-HLA-G and anti-HSP70 neutralizing mi-Abs; (g) treatment of the subject with anti-CD44v and anti-ID1/ID3 mi-Abs to neutralize circulating CSC3s from an eventual CSC3/CSC1 transition (MET), and CSC1s in niches from anchorage and self-renewal (Marangoni et al., 2009; Chen et al., 2014; Zaiden et al., 2017); (h) treatment of the subject with anti-LIF/LIFr and anti-ID1/2 mi-Abs to prevent or block CSC1 implantation in niches and angiogenesis, CSC migration by activation of the LIF-STAT3 pathway, and a possible CSC1/CSC2/CSC3 transition (EMT) (Rockman et al., 2001; American Association for Cancer Research, 2012; Guo et al., 2015; Jang et al., 2015); (i) treatment of the subject with anti-HLA-G, anti-HSP70, anti-CD44, anti-ID1/ID2/ID3 and anti-LIF/LIFr mi-Abs for general prophylaxis; (ii) final treatment with “in toto” sensitized HLA-G-free PBMCs to restore natural immunological and physiological conditions in the host.

### Considerations and Suggestions for Potential Cancer Prophylaxis

On the basis of the i-CSC/p-ESC model, it might be logically and biologically possible to use anti-HSP70/HLA-G (Qa-2 in mouse)/ID/LIF-LIFr/CD44 mi-Abs for antitumor prophylaxis. Periodical treatments with mi-Abs at defined times and in defined ways could protect healthy subjects from arising tumors (i-CSC/CSC0s) or implanting initial tumors (CSC1s). The validity of this hypothesis might be tested in appropriate animal models, inoculating before, together and after mi-Ab treatments: (a) CSCs of various human tumor types, in nude mice; (b) CSCs of various mouse tumor types in normal autologous mice. Verifying tumor development, an eventual absence of tumors with the treatment before or together with CSC inoculation would indicate a preventive effect of the mi-Abs while, with the subsequent treatment, it could also indicate a drainage effect of the mi-Abs versus initial tumors. The use of mi-Abs for periodical prophylaxis could be evaluated in relation to the subject age, in general. In particular, in women, it could be considered in relation to an eventual pregnancy, because all the mi-Ab tumor targets are crucial embryonic factors.

In conclusion, I hope that this work might make a valid contribution for a better vision of the cancer, and that it might stimulate the interest of others to debate and verify some validity of the ideas expressed, as well as to test the therapeutic potential of the suggested proposals.

## Author Contributions

The author confirms being the sole contributor of this work and has approved it for publication.

## Conflict of Interest Statement

The author declares that the research was conducted in the absence of any commercial or financial relationships that could be construed as a potential conflict of interest.
